# Hormone Therapy After Oophorectomy and Breast Cancer Risk in Women With *BRCA* Pathogenic Variant

**DOI:** 10.1001/jamanetworkopen.2026.5648

**Published:** 2026-04-08

**Authors:** Shira Regev-Sadeh, Rachel Michaelson-Cohen, Dana Madorksy-Feldman, Eitan Friedman, Shunit Armon, Amalfi Qarawani, Naama Srebnik, Joul Haddad, Vered H. Eisenberg, Yakir Segev

**Affiliations:** 1 Department of Obstetrics and Gynecology, Carmel Medical Center, Haifa, Israel; 2 School of Public Health, Gray Faculty of Medicine and Health Sciences, Tel Aviv University, Tel Aviv, Israel; 3 Ruth and Bruch Rappaport Faculty of Medicine, Technion-Israel Institute of Technology, Haifa, Israel; 4 The Fuld Family Medical Genetics Institute, Shaare Zedek Medical Center, Jerusalem, Israel; 5 The Eisenberg R&D Authority, Shaare Zedek Medical Center, Jerusalem, Israel; 6 Faculty of Medicine, The Hebrew University of Jerusalem, Jerusalem, Israel; 7 Meirav High Risk Clinic, Sheba Medical Center, Tel-Hashomer, Israel; 8 The Gray School of Medicine, Tel-Aviv University, Tel Aviv, Israel; 9 Department of Gynecology, Shaare Zedek Medical Center, Jerusalem, Israel; 10 Department of Community Medicine and Epidemiology, Lady Davis Carmel Medical Center, Haifa, Israel; 11 Ministry of Health, Tel Aviv, Israel

## Abstract

**Importance:**

Risk-reducing bilateral salpingo-oophorectomy is recommended to substantially lower ovarian cancer risk in women carrying *BRCA1* or *BRCA2* pathogenic variant (PV). The use of hormone replacement therapy (HRT) after risk-reducing bilateral oophorectomy (RRBO), although generally recommended, remains debated due to concerns about its possible role in breast cancer (BC) risk.

**Objective:**

To assess the possible association between HRT use and BC incidence after RRBO in women harboring a limited range of germline *BRCA* PVs.

**Design, Setting, and Participants:**

This retrospective multicenter cohort study was conducted at 3 medical centers, including both referral and primary care facilities, in Israel. Cancer-free women (aged ≥18 years) with *BRCA1* PV or *BRCA2* PV, with no prior mastectomy, who underwent RRBO between January 1, 2000, and December 31, 2024, and who had at least 1 year of follow-up after RRBO were included.

**Exposures:**

HRT use after RRBO.

**Main Outcomes and Measures:**

First diagnosis or incidence of invasive BC. BC diagnoses were ascertained through pathology reports and diagnostic codes used in the electronic health records; some were confirmed via participant interviews. HRT use was assessed through medical records, pharmacy dispensing data, clinic visits, and telephone interviews. Cox proportional hazards regression models, with HRT modeled as a time-varying covariate, evaluated the associations with BC risk while adjusting for potential confounders.

**Results:**

A total of 919 women (mean [SD] age at RRBO, 47.6 [8.9] years) were included, of whom 496 had *BRCA1* PV and 423 had *BRCA2* PV. During a mean (SD) follow-up of 8.8 (6.2) years, 144 women (16%) were diagnosed with invasive BC. Overall, 381 participants (42%) had ever used and 538 (58%) had never used HRT following RRBO. Ever use of HRT was not associated with increased BC risk (combined estrogen-progestin: hazard ratio [HR], 1.06 [95% CI, 0.67-1.68]; estrogen only: HR, 0.89 [95% CI, 0.48-1.63]). In duration of use analyses, each year of estrogen-only HRT was associated with a reduction in BC risk overall (HR, 0.90; 95% CI, 0.81-0.99) and a reduction among participants with *BRCA1* PV (HR, 0.87; 95% CI, 0.77-0.98).

**Conclusions and Relevance:**

In this cohort study of women with *BRCA* PV who received HRT after RRBO, estrogen-only HRT was not associated with an increased risk of BC and was associated with a lower risk of BC among women with *BRCA1* PV. Combined estrogen-progestin HRT was not associated with BC risk modification.

## Key Points

**Question:**

Is hormone replacement therapy (HRT) use after risk-reducing bilateral oophorectomy (RRBO) associated with increased breast cancer (BC) risk in women carrying *BRCA1* and *BRCA2* pathogenic variants (PVs)?

**Findings:**

In this cohort study of 919 women, HRT after RRBO was not associated with increased BC risk. Estrogen-only therapy was associated with reduced BC risk among women with *BRCA1* PV. Combined estrogen-progestin HRT was not associated with BC risk modification.

**Meaning:**

Findings of this study suggest that estrogen-only HRT after RRBO is associated with lower BC risk in women with *BRCA1* PV and does not appear to increase risk in those with *BRCA2* PV.

## Teaser

This cohort study assesses the association between hormone replacement therapy exposure and invasive breast cancer diagnosis among women in Israel carrying *BRCA1* and *BRCA2* pathogenic variants.

## Introduction

Women harboring *BRCA1* and *BRCA2* pathogenic variants (PVs) are at a considerably higher lifetime risk for developing breast and ovarian cancers.^1^ To mitigate ovarian cancer risk, National Comprehensive Cancer Network guidelines recommend risk-reducing bilateral salpingo-oophorectomy for women with *BRCA1* PV between the ages of 35 and 40 years and for women with *BRCA2* PV between 40 and 45 years of age, after completing childbirth.^2^

Risk-reducing bilateral salpingo-oophorectomy confers a substantial survival benefit among women carrying *BRCA* PVs, with a significant reduction in all-cause mortality (age-adjusted hazard ratio [HR], 0.32; 95% CI, 0.24-0.42).^3^ However, evidence from populations with average risk suggests that premature menopause may be associated with increased all-cause mortality and other long-term morbidities.^4,5,6^ Consequently, hormone replacement therapy (HRT) is frequently considered following risk-reducing bilateral oophorectomy (RRBO) to address the consequences of early surgical menopause, including vasomotor symptoms, reduced quality of life, cardiovascular morbidity, and bone loss.^7,8^ Despite its potential benefits, no international guidelines currently exist regarding use of HRT in women with *BRCA* PVs.^9,10^

The decision to initiate HRT in women with *BRCA* PVs remains complex. Although HRT can alleviate menopausal symptoms and support long-term health, concerns persist among women and their clinicians regarding a potential increase in breast cancer (BC) risk.^11^ In a cohort of 872 women with *BRCA1* PV, Kotsopoulos et al^12^ reported that estrogen-only HRT after RRBO was not associated with increased BC risk, whereas progestin-containing regimens were associated with higher risk. These findings were consistent with data from 27 347 women without *BRCA1* PV, which suggested a reduced risk associated with estrogen-only therapy (HR, 0.78; 95% CI, 0.65-0.93) and an elevated risk associated with combined estrogen-progestin regimens (HR, 1.28; 95% CI, 1.13-1.45).^13^ However, subsequent studies encompassing smaller numbers of women with *BRCA* PV have yielded inconsistent results regarding specific HRT formulations and BC risk.^14,15^

Despite existing evidence and due to the absence of international guidelines, further evaluation of the role of HRT in BC risk in women carrying *BRCA* PVis warranted. The primary objective of the current study was to assess the possible association between HRT use and BC incidence following RRBO in a large-scale, multicenter cohort of women harboring a limited range of germline *BRCA* PVs. By analyzing the implications of different HRT regimens, this study provides clinically meaningful evidence to guide therapeutic decisions and improve long-term outcomes in this high-risk population.

## Methods

### Study Design and Data Sources

We conducted a retrospective, multicenter cohort study of women harboring *BRCA1* or *BRCA2* germline PV who were followed up for at least 1 year after undergoing RRBO between January 1, 2000, and December 31, 2024. Women with a prior diagnosis of any cancer or those who underwent risk-reducing mastectomy prior to RRBO or within 1 year after RRBO were excluded. The Clalit Health Services (CHS), Shaare Zedek Medical Center, and Sheba Tel-Hashomer Medical Center institutional ethics review boards approved this study and waived the informed consent requirement because analyses were conducted on anonymized, deidentified, and unlinked data. We followed the Strengthening the Reporting of Observational Studies in Epidemiology (STROBE) reporting guideline.

Data were obtained from CHS, Shaare Zedek Medical Center, and Sheba Tel-Hashomer Medical Center in Israel. As Israel’s largest health care delivery organization, CHS serves approximately 5.4 million members (about 50% of the Israeli population)^16^ and maintains a comprehensive electronic health record (EHR) system that captures diagnoses, procedures, prescriptions, pathology reports, and longitudinal follow-up across primary and specialty care. Shaare Zedek and Sheba medical centers operate dedicated high-risk multidisciplinary clinics that provide semiannual surveillance of women with *BRCA* PVs.

At CHS, dates of diagnoses were defined as the earliest recorded date from any clinical source. At Shaare Zedek and Sheba, data were collected from participants’ medical records and interviews during follow-up visits. For participants with missing data at Shaare Zedek, information was supplemented via telephone interviews.

### Cohort Selection and Exposure Data

Eligible participants were women 18 years or older with a confirmed *BRCA1* or *BRCA2* germline PV or likely PV who had undergone RRBO and had a minimum of 1 year of postoperative follow-up. Cohort identification at all 3 centers was based on genetic test confirmation of *BRCA* variants linked to surgical procedure codes for bilateral oophorectomy with or without salpingectomy. PVs were classified as Ashkenazi Jewish founder variants or nonfounder variants. Overall, 828 women (90.1%) carried 1 of the 3 founder variants.

Follow-up began at the date of RRBO (index date). Participants were censored at the earliest occurrence of risk-reducing mastectomy, diagnosis of any malignant neoplasm other than invasive BC (excluding basal cell carcinoma), death, or end of follow-up. Administrative censoring occurred at the final visit at the high-risk clinic for participants from Shaare Tzedek and Sheba or on December 31, 2024, for participants from CHS.

The primary outcome was a first diagnosis or incidence of invasive BC among participants who were treated vs not treated with HRT after RRBO. Ductal carcinoma in situ was not included as an outcome and did not constitute an event. BC diagnoses were ascertained through pathology reports and diagnostic codes recorded in the EHR in all 3 centers. At Shaare Zedek and Sheba, completeness of BC ascertainment was further ensured through supplemental confirmation via participant interviews in cases of uncertainty.

Information on HRT use was obtained from electronic pharmacy dispensing data at CHS and from structured clinical documentation at follow-up visits in the high-risk clinics at Shaare Zedek and Sheba. Data collected included dates of HRT initiation and discontinuation, including the specific HRT formulation used. For women who changed HRT regimens during follow-up, each regimen was recorded separately according to its duration of use. Participants were classified according to HRT exposure (ever vs never), cumulative duration of HRT use (years), formulation type (estrogen only, combined estrogen-progestin, or progestin only), and route of administration (oral tablets, transdermal patch, vaginal, or topical gel or cream). Use of HRT prior to RRBO was recorded as a binary variable (yes or no). Androgen therapy was not routinely documented and therefore not included in the analysis. Additional covariates collected included prior use of hormonal levonorgestrel-releasing intrauterine device (LNG-IUD) and oral contraceptive pills (OCPs), both as binary variables; family history of BC in a first-degree relative (yes or no); and parity.

### Statistical Analysis

Baseline characteristics were summarized using descriptive statistics. Differences between HRT ever vs never users were assessed using χ^2^ tests for categorical variables and unpaired, 2-tailed *t* tests or Mann-Whitney tests for continuous variables, depending on data distribution.

To evaluate the association between HRT use and BC risk while accounting for potential confounders, we used Cox proportional hazards regression models. HRT use was modeled as a time-varying covariate to reflect changes in exposure status over time. Covariates in the multivariable models were selected based on clinical relevance and previous literature and included the following: age at RRBO, *BRCA* gene with PV, parity, prior use of OCPs, prior use of LNG-IUD, prior use of HRT, and family history of BC in a first-degree relative. Subgroup analyses were conducted by *BRCA1* PV vs *BRCA2* PV and age at RRBO (<45 vs ≥45 years). In sensitivity analyses, Cox proportional hazards regression models were repeated but excluded women with prior LNG-IUD use.

All statistical tests were 2-sided, and *P* < .05 was considered statistically significant. Data analysis was performed using IBM SPSS Statistics version 28 (IBM) and R version 4.5.2 (R Core Team).

## Results

A total of 2017 women carrying *BRCA* PVs who underwent RRBO were identified, of whom 919 (496 with *BRCA1* PV; 423 with *BRCA2* PV) met the eligibility criteria and were included in the analysis (Figure). These participants had a mean (SD) age at RRBO of 47.6 (8.9) years and a mean (SD) follow-up duration of 8.8 (6.2) years. Overall, 381 women (42%) had ever used and 538 (58%) had never used HRT following RRBO. Women who had ever used HRT were younger at the time of surgery compared with those who never did (mean [SD] age, 43.5 [7.0] vs 50.4 [9.0] years; *P* < .001). HRT users were more likely to have a history of LNG-IUD use (91 [24%] vs 61 [11%]; *P* < .001) and OCP use (295 [77%] vs 293 [55%]; *P* < .001) and were less likely to have a first-degree relative with a history of BC (176 [46%] vs 290 [54%]; *P* = .03) (Table 1).

**Figure.**
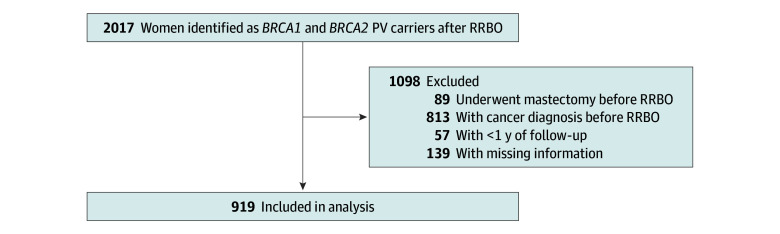
Flowchart of the Study Cohort PV, pathogenic variant; RRBO, risk-reducing bilateral oophorectomy.

**Table 1.** Characteristics of Women With *BRCA* Pathogenic Variant Stratified by Hormone Replacement Therapy Use After RRBO

Characteristic	Women, No. (%)			*P* value^a^
	Total (N = 919)	Post-RRBO HRT use		
		Never (n = 538)	Ever (n = 381)	
*BRCA* pathogenic variant				
* BRCA1*	496 (54)	275 (51)	221 (58)	.05
* BRCA2*	423 (46)	263 (49)	160 (42)	
*BRCA1* founder variant^b^	453 (49)	242 (45)	211 (55)	.002
*BRCA 2* founder variant^c^	375 (41)	231 (43)	144 (38)	.14
Age at RRBO, y				
Mean (SD)	47.6 (8.9)	50.4 (9.0)	43.5 (7.0)	<.001
≤44	403 (44)	156 (29)	247 (65)	
45-49	221 (24)	130 (24)	91 (24)	
≥50	295 (32)	252 (47)	43 (11)	
Age at genetic testing, mean (SD), y	45.6 (11.5)	49.6 (10.8)	39.9 (9.9)	<.001
Parity				
0	96 (10)	66 (12)	30 (8)	.08
1-3	590 (64)	343 (64)	247 (65)	
≥4	233 (25)	129 (24.0)	104 (27)	
OCP use	588 (64)	293 (55)	295 (77)	<.001
LNG-IUD use^d^	152 (17)	61 (11)	91 (24)	<.001
HRT use before RRBO	178 (20)	89 (17)	89 (23)	.01
Family history of BC	466 (51)	290 (54)	176 (46)	.03
Incident BC	144 (16)	101 (19)	43 (11)	.002

Abbreviations: BC, breast cancer; HRT, hormone replacement therapy; LNG-IUD, levonorgestrel-releasing intrauterine device; OCP, oral contraceptive pill; RRBO, risk-reducing bilateral oophorectomy.

^a^ *P* values were calculated using unpaired, 2-tailed *t* test for continuous variables and χ^2^ test for categorical variables. All tests were 2-sided.

^b^ *BRCA1* c.68_69delAG or *BRCA1* c.5266dupC.

^c^ *BRCA2* c.5946delT.

^d^ LNG-IUD used as a contraceptive prior to RRBO.

Among HRT users, the mean (SD) duration of HRT after RRBO was 5.09 (3.83) years. The most commonly used formulation was combined estrogen-progestin (256 [68%]), while 119 women (31%) used estrogen-only therapy (Table 2). The most common route of administration was oral tablets (205 [54%]), followed by transdermal patches (71 [19%]) and mixed regimens (65 [17%]) (Table 2). Overall, 82 participants (22%) were still taking HRT at age 50 years, 34 participants (9%) were still taking it at age 55 years, and 33 participants (9%) were still taking it up to or beyond age 60 years.

**Table 2.** Characteristics of Participants Receiving Hormone Replacement Therapy: Route of Administration and Hormone Formulation

Characteristic	Women with HRT use, No. (%) (n = 381)	Duration of use, median (IQR), y
Dominant route of administration^a^^,^^b^		
Oral tablets	205 (54)	4.33 (2.00-7.28)
Transdermal patch	71 (19)	3.50 (1.79-7.08)
Vaginal	36 (9)	4.82 (1.86-6.78)
Topical gel or cream	3 (1)	2.00 (1.50-3.02)
Mixed regimens^c^	65 (17)	4.59 (2.58-8.00)
HRT formulation^a^		
Estrogen only	119 (31)	3.42 (1.39-7.21)
Combined estrogen-progestin	256 (68)	4.56 (2.35-7.30)
Progestin only	3 (1)	5.00 (2.97-5.32)
Age at HRT initiation, y	NA	42.21 (40.00-46.84)
Time since RRBO to HRT use, mo	NA	0.78 (0-8.18)

Abbreviations: HRT, hormone replacement therapy; NA, not applicable; RRBO, risk-reducing bilateral oophorectomy.

^a^ Each woman categorized based on the HRT regimen used for the longest cumulative duration.

^b^ Total number was 380, as information on route of administration was missing for 1 participant.

^c^ Mixed regimens used simultaneously.

There were 144 women (16%) diagnosed with invasive BC in the cohort. Table 3 presents univariate and multivariable HRs for BC after RRBO. Multivariable HRs were also calculated by *BRCA1* PV and *BRCA2* PV separately and by age at RRBO (<45 vs ≥45 years). No significant difference in BC risk was observed between women who ever used HRT and those who never did. This finding was consistent for both combined estrogen-progestin therapy (HR, 1.06; 95% CI, 0.67-1.68; *P* = .80) and estrogen-only therapy (HR, 0.89; 95% CI, 0.48-1.63; *P* = .70). BC risk was lower in women with *BRCA2* PV vs *BRCA1* PV (HR, 0.46; 95% CI, 0.32-0.65; *P* < .001). HRT use remained unassociated with BC risk across all groups.

**Table 3.** Hormone Replacement Therapy Use After RRBO and Breast Cancer Risk in Women With *BRCA1* or *BRCA2* Pathogenic Variant

Variable	Incident BC, No. (%)	Univariate HR (95% CI)	*P* value	Multivariable HR (95% CI)	*P* value
**Ever or never HRT use after RRBO and BC risk (n = 919)**					
*BRCA1* pathogenic variant	97 (11)	1 [Reference]	NA	1 [Reference]	NA
*BRCA2* pathogenic variant	47 (5)	0.47 (0.33-0.67)	<.001	0.46 (0.32-0.65)	<.001
Age at RRBO, y					
≤44	NA	1 [Reference]	NA	1 [Reference]	NA
45-49	NA	1.12 (0.75-1.7)	.58	1.26 (0.82-1.93)	.29
≥50	NA	1.06 (0.72-1.55)	.77	1.26 (0.80-1.98)	.32
Parity					
0	NA	1 [Reference]	NA	1 [Reference]	NA
1-3	NA	1.27 (0.71-2.25)	.42	1.22 (0.67-2.2)	.52
≥4	NA	0.91 (0.47-1.74)	.77	0.88 (0.44-1.75)	.71
OCP use	NA	1.06 (0.76-1.48)	.74	1.09 (0.74-1.61)	.66
LNG-IUD use^a^	NA	1.47 (0.97-2.22)	.07	1.69 (1.09-2.61)	.02
HRT use before RRBO	NA	1.11 (0.74-1.68)	.61	0.94 (0.58-1.53)	.80
Family history of BC	NA	1.09 (0.78-1.51)	.63	0.99 (0.69-1.41)	.94
HRT use after RRBO: estrogen only	12 (13)	0.97 (0.54-1.76)	.93	0.89 (0.48-1.63)	.70
HRT use after RRBO: combined estrogen-progestin	31 (3)	1.06 (0.71-1.60)	.77	1.06 (0.67-1.68)	.80
**HRT use after RRBO and BC risk stratified by *BRCA* pathogenic variant**					
*BRCA1* (n = 496)^b^					
Never	63 (13)	1 [Reference]	NA	1 [Reference]	NA
Ever: estrogen only	10 (2)	1.06 (0.55-2.04)	.86	1.03 (0.52-2.04)	.94
Ever: combined estrogen-progestin	24 (5)	1.10 (0.69-1.77)	.69	1.14 (0.66-1.97)	.64
LNG-IUD use^a^	20 (8)	1.94 (1.18-3.18)	.01	2.00 (1.18-3.36)	.01
*BRCA2* (n = 423)^b^					
Never	38 (9)	1 [Reference]	NA	1 [Reference]	NA
Ever: estrogen only	2 (1)	0.58 (0.14-2.39)	.50	0.69 (0.16-2.98)	.60
Ever: combined estrogen-progestin	7 (2)	0.84 (0.37-1.88)	.70	0.84 (0.35-2.03)	.70
LNG-IUD use^a^	8 (2)	1.09 (0.51-2.34)	.80	1.26 (0.55-2.90)	.60
**HRT use after RRBO and BC risk stratified by age at RRBO** ^a^					
Age at RRBO <45 y (n = 445)^c^					
Never	33 (7)	1 [Reference]	NA	1 [Reference]	NA
Ever: estrogen only	5 (1)	0.95 (0.38-2.36)	.91	0.94 (0.36-2.46)	.91
Ever: combined estrogen-progestin	26 (6)	1.35 (0.81-2.26)	.25	1.27 (0.73-2.22)	.40
Age at RRBO ≥45 y (n = 474)^c^					
Never	68 (14)	1 [Reference]	NA	1 [Reference]	NA
Ever: estrogen only	7 (1)	0.98 (0.45-2.14)	.91	0.78 (0.35-1.77)	.56
Ever: combined estrogen-progestin	5 (1)	0.56 (0.20-1.52)	.25	0.51 (0.18-1.45)	.20

Abbreviations: BC, breast cancer; HR, hazard ratio; HRT, hormone replacement therapy; LNG-IUD, levonorgestrel-releasing intrauterine device; NA, not applicable; OCP, oral contraceptive pill; RRBO, risk-reducing bilateral oophorectomy.

^a^ LNG-IUD used as a contraceptive prior to RRBO.

^b^ Models adjusted for age at RRBO, parity, OCP use, LNG-IUD use, HRT use before RRBO, and family history of BC.

^c^ Models adjusted for *BRCA* pathogenic variant, parity, OCP use, LNG-IUD use, HRT use before RRBO, and family history of BC.

LNG-IUD use prior to RRBO was associated with a significantly increased BC risk (HR, 1.69; 95% CI, 1.09-2.61; *P* = .02), particularly among women with *BRCA1* PV (HR, 2.00; 95% CI, 1.18-3.36; *P* = .01). The association was not statistically significant among women with *BRCA2* PV (HR, 1.26; 95% CI, 0.55-2.90; *P* = .60).

The association between duration of HRT use after RRBO and BC risk is shown in Table 4. For the entire study population, each year of estrogen-only HRT was associated with a reduction in BC risk (HR, 0.90; 95% CI, 0.81-0.99; *P* = .04), whereas combined estrogen-progestin HRT showed no significant association (HR, 0.95; 95% CI, 0.89-1.01; *P* = .07). When stratified by *BRCA* PV, each year of estrogen-only HRT was associated with a reduction in BC risk among women with *BRCA1* PV (HR, 0.87; 95% CI, 0.77-0.98; *P* = .02) but was not significantly associated with BC risk in women with *BRCA2* PV (HR, 0.96; 95% CI, 0.82-1.12; *P* = .57). Combined estrogen-progestin HRT was not significantly associated with BC risk in women with either *BRCA1* PV or *BRCA2* PV. For participants who underwent RRBO before age 45 years, long-term use of combined estrogen-progestin HRT (≥5 years) was associated with an increased risk of BC in univariate analysis (HR, 2.38; 95% CI, 1.03-5.50; *P* = .04) but was not statistically significant after multivariable adjustment (HR 2.31; 95% CI, 0.99-5.40; *P* = .05). For participants who underwent RRBO at age 45 years or older, HRT use was not associated with a modified BC risk for either estrogen-only or combined estrogen-progestin HRT (Table 4). Analyses excluding women with prior LNG-IUD use are shown for ever vs never HRT use and duration of HRT use in eTables 1 and 2 in Supplement 1.

**Table 4.** Duration of Hormone Replacement Therapy Use After RRBO and Breast Cancer Risk in Women With *BRCA1* or *BRCA2* Pathogenic Variant

Variable	Univariate HR per year of HRT use (95% CI)	*P* value	Multivariable HR per year of HRT use (95% CI)	*P* value
**HRT use after RRBO and BC risk (n = 919)** ^a^				
Estrogen only				
Never	1 [Reference]	NA	1 [Reference]	NA
Ever, per y	0.92 (0.82-1.02)	.18	0.90 (0.81-0.99)	.04
0-2	1.28 (0.59-2.80)	.53	1.16 (0.53-2.50)	.71
2-5	0.99 (0.31-3.19)	.99	0.97 (0.30-3.10)	.96
≥5	0.51 (0.13-2.10)	.35	0.48 (0.12-1.95)	.30
Combined estrogen-progestin				
Never	1 [Reference]	NA	1 [Reference]	NA
Ever, per y	0.96 (0.91-1.02)	.11	0.95 (0.89-1.01)	.07
0-2	1.03 (0.51-2.07)	.94	1.03 (0.50-2.11)	.94
2-5	0.90 (0.46-1.78)	.77	0.87 (0.43-1.74)	.68
≥5	1.40 (0.73-2.68)	.32	1.44 (0.73-2.86)	.29
**HRT use after RRBO and BC risk stratified by *BRCA* pathogenic variant**				
*BRCA1* (n = 496)				
Estrogen only				
Never	1 [Reference]	NA	1 [Reference]	NA
Ever, per y^b^	0.90 (0.77-1.04)	.15	0.87 (0.77-0.98)	.02
0-2^c^	1.50 (0.68-3.30)	.32	1.59 (0.72-3.50)	.25
2-5^c^	0.96 (0.23-4.00)	.96	1.02 (0.24-4.28)	.98
≥5^c^	0.40 (0.05-2.89)	.36	0.41 (0.06-3.02)	.38
Combined estrogen-progestin				
Never	1 [Reference]	NA	1 [Reference]	NA
Ever, per y^b^	0.94 (0.88-1.01)	.11	0.93 (0.87-1.00)	.04
0-2^c^	0.98 (0.42-2.27)	.95	1.03 (0.43-2.45)	.95
2-5^c^	1.24 (0.61-2.52)	.55	1.27 (0.61-2.65)	.52
≥5^c^	1.31 (0.58-2.97)	.52	1.34 (0.58-3.07)	.49
*BRCA2* (n = 423)				
Estrogen only				
Never	1 [Reference]	NA	1 [Reference]	NA
Ever, per y^b^	0.94 (0.80-1.10)	.44	0.96 (0.82-1.12)	.57
0-2^c^	NA	NA	NA	NA
2-5^c^	1.00 (0.13-7.53)	.99	1.01 (0.13-7.61)	.99
≥5^c^	0.67 (0.09-5.00)	.70	0.67 (0.09-5.02)	.70
Combined estrogen-progestin				
Never	1 [Reference]	NA	1 [Reference]	NA
Ever, per y^b^	0.97 (0.88-1.08)	.62	0.99 (0.88-1.10)	.80
0-2^c^	1.12 (0.32-3.92)	.86	1.07 (0.30-3.85)	.92
2-5^c^	NA	NA	NA	NA
≥5^c^	1.42 (0.49-4.12)	.52	1.33 (0.43-4.08)	.62
**HRT use after RRBO and BC risk stratified by age at RRBO**				
Age at RRBO <45 y (n = 445)				
Estrogen only				
Never	1 [Reference]	NA	1 [Reference]	NA
Ever, per y^d^	0.92 (0.78-1.09)	.34	0.90 (0.74-1.11)	.33
0-2^e^	1.11 (0.33-3.73)	.87	1.15 (0.34-3.92)	.83
2-5^e^	0.62 (0.08-4.71)	.65	0.58 (0.08-4.41)	.60
≥5^e^	1.03 (0.13-7.80)	.98	1.15 (0.15-8.88)	.89
Combined estrogen-progestin				
Never	1 [Reference]	NA	1 [Reference]	NA
Ever, per y^d^	0.97 (0.90-1.04)	.36	0.95 (0.88-1.02)	.14
0-2^e^	0.93 (0.38-2.29)	.88	0.88 (0.35-2.18)	.78
2-5^e^	1.18 (0.54-2.60)	.67	1.05 (0.48-2.32)	.91
≥5^e^	2.38 (1.03-5.50)	.04	2.31 (0.99-5.40)	.05
Age at RRBO ≥45 y (n = 474)				
Estrogen only				
Never	1 [Reference]	NA	1 [Reference]	NA
Ever, per y^d^	0.91 (0.78-1.05)	.20	0.89 (0.78-1.01)	.06
0-2^e^	1.40 (0.51-3.89)	.87	1.24 (0.45-3.42)	.68
2-5^e^	1.26 (0.31-5.23)	.75	1.38 (0.33-5.77)	.66
≥5^e^	0.35 (0.05-2.52)	.30	0.34 (0.05-2.50)	.29
Combined estrogen-progestin				
Never	1 [Reference]	NA	1 [Reference]	NA
Ever, per y^d^	0.93 (0.81-1.07)	.33	0.92 (0.79-1.07)	.27
0-2^e^	1.33 (0.40-4.44)	.65	1.44 (0.42-4.86)	.56
2-5^e^	NA	NA	NA	NA
≥5^e^	0.82 (0.20-3.37)	.78	0.70 (0.17-2.92)	.63

Abbreviations: BC, breast cancer; HR, hazard ratio; HRT, hormone replacement therapy; NA, not applicable; RRBO, risk-reducing bilateral oophorectomy.

^a^ Models adjusted for *BRCA* pathogenic variant, age at RRBO, parity, oral contraceptive pill (OCP) use, levonorgestrel-releasing intrauterine device (LNG-IUD) use, HRT use before RRBO, and family history of BC.

^b^ Models adjusted for age at RRBO, parity, OCP use, LNG-IUD use, HRT use before RRBO, and family history of BC.

^c^ Models adjusted for age as a categorical variable (<45 or ≥45 years) and LNG-IUD use.

^d^ Models adjusted for *BRCA* pathogenic variant, parity, OCP use, LNG-IUD use, HRT use before RRBO, and family history of BC.

^e^ Models adjusted for *BRCA* PV and LNG-IUD use.

## Discussion

In this multicenter cohort study of 919 participants with *BRCA* PV, HRT use after RRBO was not associated with an increased risk of BC. Moreover, estrogen-only HRT showed possible protective benefit in women with *BRCA1* PV. In the duration analysis, each year of estrogen-only HRT was associated with a significant 10% reduction in BC risk. Among those carrying *BRCA1* PV, estrogen-only HRT was associated with a 13%-reduction per year of use. In contrast, combined estrogen-progestin HRT was not consistently associated with either increased or decreased BC risk in women with either *BRCA1* PV or *BRCA2* PV.

Our findings are consistent with the largest study to date by Kotsopoulos et al,^12^ which included 872 women with *BRCA1* PV with a mean follow-up of 7.6 years. The authors reported an 8% reduction in BC risk per year of estrogen-containing HRT (HR, 0.92; 95% CI, 0.83-1.01) and no association for progestin-containing HRT (HR, 1.08; 95% CI, 0.92-1.27).^12^ The current study included a larger cohort with longer follow-up and a limited spectrum of germline PVs, reducing genetic heterogeneity and potential confounding. Additionally, more comprehensive adjustment is provided by accounting for prior LNG-IUD use and prior OCP use. Our analyses were restricted to women with at least 1 year of follow-up after RRBO, ensuring that the observed associations more accurately reflect the implications of post-RRBO HRT exposure.

Our results also align with evidence in the general population. Long-term follow-up of the Women’s Health Initiative randomized clinical trial demonstrated lower incidence of invasive BC among women receiving estrogen-only HRT (HR, 0.78; 95% CI, 0.65-0.93), whereas combined estrogen-progestin treatment was associated with an increased BC risk (HR, 1.28; 95% CI, 1.13-1.45).^13^

Differential associations between HRT use and BC risk by age at RRBO were not consistently observed. Among participants who underwent RRBO before age 45 years, long-term use of combined estrogen-progestin HRT (≥5 years) was associated with an increased BC risk in univariate analysis; this association remained borderline after multivariable adjustment (HR, 2.31; 95% CI, 0.99-5.40). In contrast, HRT was not associated with BC risk modification among women who underwent RRBO at 45 years or older. These findings differ from prior reports suggesting increased BC risk among women older than 45 years at surgery.^12,14^

We found an association between prior LNG-IUD use before RRBO and an increased risk of BC in the overall cohort (HR, 1.69; 95% CI, 1.09-2.61), particularly among women with *BRCA1* PV (HR, 2.00; 95% CI, 1.18-3.36). In the general population, large-scale studies and meta-analyses have reported a 13% to 32% relative increase in BC risk among ever-users of LNG-IUD, especially with current use.^17,18,19^ In women with *BRCA* PV, the association between LNG-IUD use and BC risk is less established. Recent pooled prospective cohort data from 4 prospective studies, encompassing 3882 women with *BRCA1* PV and 1509 women with *BRCA2* PV, reported that ever-use of hormonal contraceptives (including progestin-only methods but not hormonal IUDs) was associated with an increased BC risk in women with *BRCA1* PV (HR, 1.29; 95% CI, 1.04-1.60), whereas no such association was found among women with *BRCA2* PV.^20^ In the current study, prior use of OCPs, regardless of formulation, was not associated with BC risk modification in either the overall or *BRCA*-stratified analyses.

*BRCA1*-related BCs are predominantly triple-negative BCs and are believed to be promoted by progestin-driven activation of the RANK/RANKL (receptor activator of NF-κB/ligand) pathway, which stimulates mammary epithelial proliferation.^21,22^ Estrogen-only HRT avoids this protumorigenic pathway and may exert protection through proapoptotic and antiproliferative properties.^23,24^ In the Women’s Health Initiative trial, estrogen-only HRT was associated with lower BC incidence and mortality compared with placebo, whereas the addition of a progestin increased incidence, consistent with this mechanism.^13,25^ This biologic rationale provides plausibility for the observed association between estrogen-only HRT use and lower BC risk among women carrying *BRCA1* PV, which is consistent with prior *BRCA1*-specific cohort data.^12^ Thus, our findings highlight the different implications of estrogen-only vs progestin-only hormonal exposures in women with *BRCA1* PV and emphasize the need for individualized counseling regarding both HRT and contraceptive choices in this high-risk group.

*BRCA2*-related BCs are more commonly estrogen receptor–positive BCs,^26^ yet current evidence does not indicate a unique sensitivity to exogenous estrogen or progestin exposure in this population, unlike the RANK/RANKL-driven proliferation described in women with *BRCA1* PV.^24,27^ In our cohort, neither estrogen-only HRT nor combined estrogen-progestin HRT was associated with modification of BC risk among women with *BRCA2* PV. While use of progestin-only contraception (LNG-IUD) was associated with an increased BC risk in the overall study population, it was not specifically associated with significantly increased risk in women with *BRCA2* (HR, 1.26; 95% CI, 0.55-2.9), possibly reflecting limited statistical power due to a smaller effect size in this population compared with those with *BRCA1*.

The most common routes of HRT administration in this study were oral pills, followed by transdermal patches. Although we collected detailed data on formulations and routes, this study lacked statistical power to stratify the analysis by these subgroups. Evidence from the general population suggests that systemic absorption varies significantly by route and formulation,^28^ which could affect both the magnitude and direction of risk. Larger studies are needed to clarify whether specific HRT formulations confer differential implications for BC risk in women with *BRCA* PV.

To our knowledge, this study is the largest multicenter analysis of the association between HRT use and BC risk in women carrying both *BRCA1* or *BRCA2* PVs following RRBO. Clinically, the findings are important, as systemic estrogen-only regimens are appropriate only for women who have undergone a hysterectomy. Decisions regarding concomitant hysterectomy at the time of RRBO should therefore be considered in the context of anticipated HRT needs, particularly for women who did not undergo a mastectomy. The results highlight the importance of multidisciplinary counseling to support shared, individualized decision-making for women with *BRCA1* and *BRCA2* PVs and indicate the differences between these populations. Future studies with larger cohorts are needed to evaluate the impact of specific hormonal formulations, doses, and routes of administration as well as their long-term safety in this high-risk population.

### Strengths and Limitations

Study strengths include its large, multicenter design; the relatively long mean follow-up of 8.8 years; and access to detailed exposure data, including dates of initiation and cessation, duration of treatment, specific formulations, and routes of administration. Extensive information was collected on potential confounders, obtained through direct participant questioning and supplemented with telephone interviews, EHR data, and pharmacy dispensing data.

This study also has several limitations, including its observational design, which may leave residual confounding despite multivariable adjustment. Since HRT is not always initiated immediately following RRBO, the study is susceptible to immortal time bias; however, this bias was addressed by modeling HRT use as a time-dependent covariate. Recall bias related to self-reported exposure data is possible. Finally, missing data led to the exclusion of a subset of participants, reducing the sample size and potentially introducing selection bias toward women more engaged with their medical care.

## Conclusions

In this cohort study of women harboring *BRCA* PV after RRBO, estrogen-only HRT after the surgical procedure was not associated with an increased risk of BC and was associated with a reduced risk in women with *BRCA1* PV. Combined estrogen-progestin HRT was not consistently associated with BC risk modification. In contrast, prior use of progestin-only contraception (LNG-IUD) was associated with increased BC risk among women with *BRCA1* PV, potentially mediated through stimulation of the RANK/RANKL pathway.

## Notes

Supplement 1. **eTable 1.** HRT Use After RRBO and Breast Cancer Risk in Women With BRCA1 and BRCA2 Pathogenic Variant, Excluding Patients With Prior LNG-IUD Use **eTable 2.** Duration of HRT Use After RRBO and Breast Cancer Risk in Women With BRCA1 and BRCA2 Pathogenic Variant, Excluding Patients With Prior LNG-IUD Use

Supplement 2.
Data Sharing Statement
