# Supplementary material for: Hormone Therapy After Oophorectomy and Breast Cancer Risk in Women With BRCA Pathogenic Variant
Source: JAMA Netw Open. 2026 Apr 8;9(4):e265648. doi: 10.1001/jamanetworkopen.2026.5648 (PMC13063081; doi:10.1001/jamanetworkopen.2026.5648)
Supplement: Supplement 1. — eTable 1. HRT Use After RRBO and Breast Cancer Risk in Women With BRCA1 and BRCA2 Pathogenic Variant, Excluding Patients With Prior LNG-IUD Use eTable 2. Duration of HRT Use After RRBO and Breast Cancer Risk in Women With BRCA1 and BRCA2 Pathogenic Variant, Excluding Patients With Prior LNG-IUD Use [file jamanetwopen-e265648-s001.pdf]

Supplemental Online Content

Regev-Sadeh S, Michaelson-Cohen R, Madorksy-Feldman D, et al. Hormone therapy after oophorectomy and breast cancer risk in women with *BRCA* pathogenic variant. *JAMA Netw Open*. 2026;9(4):e265648. doi:10.1001/jamanetworkopen.2026.5648

eTable 1. HRT Use After RRBO and Breast Cancer Risk in Women With BRCA1 and BRCA2 Pathogenic Variant, Excluding Patients With Prior LNG-IUD Use

eTable 2. Duration of HRT Use After RRBO and Breast Cancer Risk in Women With BRCA1 and BRCA2 Pathogenic Variant, Excluding Patients With Prior LNG-IUD Use

This supplemental material has been provided by the authors to give readers additional information about their work.

© 2026 Regev-Sadeh S et al. *JAMA Network Open*

eTable 1. HRT Use After RRBO and Breast Cancer Risk in Women With BRCA1 and BRCA2 Pathogenic Variant, Excluding Patients With Prior LNG-IUD Use

Variable	Univariate HR (95% CI)	p-value	Multivariable HR (95% CI)	p-value
**S1A: Ever/never HRT use after RRBO and breast cancer risk (n=767)**				
BRCA1 pathogenic variant	1 [Reference]	NA	1 [Reference]	NA
BRCA2 pathogenic variant	0.55 (0.37-0.81)	0.002	0.52 (0.35-0.76)	0.001
Age at RRBO				
<=44	1 [Reference]	NA	1 [Reference]	NA
45-49	1.52 (0.95-2.42)	0.08	1.65 (1.02-2.65)	0.04
>=50	1.19 (0.76-1.87)	0.44	1.38 (0.83-2.30)	0.21
Parity				
0	1 [Reference]	NA	1 [Reference]	NA
1-3	1.24 (0.68-2.28)	0.49	1.22 (0.66-2.28)	0.52
4+	1.16 (0.57-2.39)	0.68	1.10 (0.53-2.30)	0.80
OCP use	1.10 (0.74-1.63)	0.63	1.10 (0.72-1.68)	0.67
HRT before RRBO	1.00 (0.62-1.64)	0.99	1.03 (0.62-1.71)	0.92
Family history of breast cancer	1.04 (0.70-1.54)	0.86	1.07 (0.72-1.58)	0.76
HRT use post-RRBO (E only)	0.77 (0.37-1.58)	0.47	0.72 (0.35-1.51)	0.39
HRT use post-RRBO (E+P)	0.95 (0.58-1.58)	0.85	0.96 (0.56-1.66)	0.89
**S1B: HRT use after RRBO and breast cancer risk stratified by BRCA pathogenic variant**				
**BRCA1 (n=423)**[1]				
Never	1 [Reference]	NA	1 [Reference]	NA
Ever, E only	0.87 (0.40-1.90)	0.72	0.88 (0.40-1.97)	0.76
Ever, E+P	0.98 (0.55-1.76)	0.95	1.07 (0.56-2.03)	0.83
**BRCA2 (n=344)**[1]				
Never	1 [Reference]	NA	1 [Reference]	NA
Ever, E only	0.36 (0.05-2.64)	0.31	0.44 (0.06-3.31)	0.42
Never, E+P	0.66 (0.23-1.90)	0.45	0.72 (0.24-2.13)	0.55
**S1C: HRT use after RRBO and breast cancer risk stratified by age at RRBO**[1]				
**Age at RRBO < 45 (n=363)**[2]				
Never	1 [Reference]	NA	1 [Reference]	NA
Ever, E only	0.69 (0.21-2.22)	0.53	0.65 (0.20-2.18)	0.49
Ever, E+P	1.21 (0.65-2.26)	0.55	1.21 (0.63-2.31)	0.57
**Age at RRBO ≥ 45 (n=404)**[2]				
Never	1 [Reference]	NA	1 [Reference]	NA
Ever, E only	0.81 (0.32-2.03)	0.66	0.71 (0.28-1.80)	0.47
Ever, E+P	0.6 (0.19-1.92)	0.39	0.53 (0.16-1.74)	0.30

© 2026 Regev-Sadeh S et al. *JAMA Network Open*

Abbreviations: RRBO, risk-reducing bilateral oophorectomy; HRT, hormone replacement therapy; HR, hazard ratio; CI, confidence interval; LNG-IUD, levonorgestrel-releasing intrauterine device; OCP, oral contraceptive pills; E, estrogen; E+P, estrogen plus progestin.

[1]Models adjusted for age at RRBO, parity, oral contraceptive use, HRT before RRBO and family history of breast cancer

[2]Models adjusted for BRCA pathogenic variant, parity, oral contraceptive use, HRT before RRBO and family history of breast cancer

© 2026 Regev-Sadeh S et al. *JAMA Network Open*

eTable 2. Duration of HRT Use After RRBO and Breast Cancer Risk in Women With BRCA1 and BRCA2 Pathogenic Variant, Excluding Patients With Prior LNG-IUD Use

Variable	Univariate HR per year of HRT use (95% CI)	p-value	Multivariable HR per year of HRT use (95% CI)	p-value
**S2A: HRT use after RRBO and breast cancer risk (n=767)[1]**				
Never	1 [Reference]	NA	1 [Reference]	NA
E only, per year	0.88 (0.76-1.02)	0.08	0.87 (0.75-1.01)	0.08
E+P, per year	0.95 (0.88-1.02)	0.14	0.94 (0.87-1.01)	0.09
**S2B: HRT use after RRBO and breast cancer risk stratified by BRCA pathogenic variant**				
**BRCA1 (n=423)[2]**				
Never	1 [Reference]	NA	1 [Reference]	NA
E only, per year	0.82 (0.70-0.96)	0.02	0.81 (0.69-0.96)	0.01
E+P, per year	0.94 (0.87-1.03)	0.18	0.94 (0.86-1.03)	0.18
**BRCA2 (n=344)[2]**				
Never	1 [Reference]	NA	1 [Reference]	NA
E only, per year	0.95 (0.78-1.15)	0.59	0.96 (0.79-1.17)	0.70
E+P, per year	0.90 (0.76-1.06)	0.21	0.92 (0.78-1.09)	0.32
**S3C: HRT use after RRBO and breast cancer risk stratified by BRCA pathogenic variant**				
**Age at RRBO < 45 (n=363)[3]**				
Never	1 [Reference]	NA	1 [Reference]	NA
E only, per year	0.93 (0.77-1.11)	0.41	0.91 (0.74-1.11)	0.35
E+P, per year	0.96 (0.87-1.05)	0.40	0.96 (0.88-1.05)	0.36
**Age at RRBO ≥ 45 (n=404)[3]**				
Never	1 [Reference]	NA	1 [Reference]	NA
E only, per year	0.83 (0.96-0.98)	0.03	0.81 (0.67-0.98)	0.03
E+P, per year	0.90 (0.73-1.11)	0.33	0.87 (0.70-1.10)	0.25

Abbreviations: RRBSO, risk-reducing bilateral salpingo-oophorectomy; HRT, hormone replacement therapy; HR, hazard ratio; CI, confidence interval; LNG-IUD, levonorgestrel-releasing intrauterine device; E, estrogen; E+P, estrogen plus progestin.

[1]Model adjusted for BRCA PV, age at RRBSO, parity, oral contraceptive use, HRT before RRBSO and family history of breast cancer

[2]Models adjusted for age at RRBSO, parity, oral contraceptive use, HRT before RRBSO and family history of breast cancer

[3]Models adjusted for BRCA pathogenic variant, parity, oral contraceptive use, HRT before RRBSO and family history of breast cancer

© 2026 Regev-Sadeh S et al. *JAMA Network Open*
