# Supplementary material for: Hormone Therapy After Oophorectomy and Breast Cancer Risk in Women With BRCA Pathogenic Variant
Source: JAMA Netw Open. 2026 Apr 8;9(4):e265648. doi: 10.1001/jamanetworkopen.2026.5648 (PMC13063081; doi:10.1001/jamanetworkopen.2026.5648)
Supplement: Supplement 2. — Data Sharing Statement [file jamanetwopen-e265648-s002.pdf]

Data Sharing Statement

Regev-Sadeh. Hormone Therapy After Oophorectomy and Breast Cancer Risk in Women With BRCA Pathogenic Variant. *JAMA Netw Open*. Published April 08, 2026.
doi:10.1001/jamanetworkopen.2026.5648

Data
Data available: No

Additional Information
Explanation for why data not available: The data include sensitive patient-level information from medical records and cannot be shared due to privacy and institutional restrictions.
